# Impact of Time to Initiation of Treatment on the Quality of Life of Women with Breast Cancer

**DOI:** 10.3390/ijerph17228325

**Published:** 2020-11-11

**Authors:** Magdalena Konieczny, Elżbieta Cipora, Wojciech Roczniak, Magdalena Babuśka-Roczniak, Marek Wojtaszek

**Affiliations:** Medical Institute, The Jan Grodek State University in Sanok, 38-500 Sanok, Poland; elacipora@interia.pl (E.C.); wojciech_roczniak@interia.pl (W.R.); magda_babuska@vp.pl (M.B.-R.); wojtaaszek@gmail.com (M.W.)

**Keywords:** breast cancer, quality of life, treatment initiation, time

## Abstract

Introduction: Breast cancer is the most common malignancy in women. Due to the large number of women living with breast cancer and the increasing incidence of this cancer, it is very important to understand the factors determining the quality of life (QOL) of patients. The aim of the study. The aim of the study was to determine the impact of time to initiation of treatment on the quality of life of women with breast cancer. Materials and methods. The study involved 324 women with breast cancer, treated at the Podkarpackie Oncology Centre in Brzozów, Poland. The study was conducted using a diagnostic survey, using a standardised questionnaire to measure the quality of life of women treated for breast cancer, i.e., the European Organization for Research and Treatment of Cancer Quality of Life Questionnaire (EORTC) QLQ-C30 and the QLQ-BR23 module, as well as a proprietary survey questionnaire. Statistical analysis was performed using the Statistica 10.0 software (StatSoft Inc., 2011). A *p* value of <0.05 was considered statistically significant. Results: The examined women had a reduced overall quality of life and health (M = 53.88). The quality of life was higher in women who consulted a doctor the earliest after noticing initial symptoms of the disease, i.e., up to one week (M = 57.58), compared to patients who delayed the decision (over four weeks; M = 47.8) (*p =* 0.002). The quality of life was also considered higher by women who received treatment within two weeks of diagnosis (M = 56.79) and was lower for patients who waited for treatment for more than two months (M = 43.68). Statistically significant relationships were demonstrated for functional scales and disease intensity. Conclusions: Women diagnosed with breast cancer had a considerably lower overall quality of life. A relatively higher quality of life was experienced by patients who consulted a doctor the earliest after discovering symptoms of the disease and those whose waiting time for treatment was shorter. In a systematic manner, the individual stages of diagnosis should be maximally reduced and breast cancer treatment initiated without delay.

## 1. Introduction

Breast cancer is the most frequently diagnosed malignancy in women. Every year, about 1.7 million women are diagnosed with breast cancer worldwide [1,2]. In Poland, breast cancer is also a very serious health, social and economic issue, and currently about 20,000 new cases are diagnosed every year. Incidence rates for this type of cancer have increased in Poland from 200 to 340 per 100,000 women per year [3]. It is estimated that this number will increase in the coming years. Consequently, cancer therapy should be adapted towards treating both the patient’s body and mind. With such a holistic approach, the goal of treatment is not only to extend the life of the ill person, but also to improve their quality of life [4].

Because of a steady increase in the number of cases of breast cancer, but also higher five-year survival rates, which are being achieved through, among others, the implementation of preventative programs and advances in treatment, it becomes evident that an adequate quality of life for patients should be ensured [5]. For these reasons, it is important to recognize the factors affecting quality of life. Medical literature, especially from international sources, provide results of research on determinants affecting the quality of life of women with breast cancer. It was found, among others, correlation between sociodemographic factors, the type of treatment undertaken, and the quality of life of women with breast cancer [6,7,8]. The research also presents information on the quality of life of women after treatment [9]. In Poland, studies assessing the quality of life of cancer patients are not sufficient. Although studies evaluating the quality of life of women with breast cancer have been conducted taking into account demographic, social and medical factors (type of treatment), there are few publications in this field [10,11,12].

It should be emphasized, that in both the domestic and international literature, there are no reports specifying the relationship between the quality of life of women with breast cancer and the time of the first medical assessment by a doctor and the waiting time of the subsequent implementation of oncological treatment. The results of such studies are particularly important in the context of delays in diagnosis and breast cancer treatment [13].

The aim of the study was to assess the quality of life of women with breast cancer, based on the time to initial treatment.

## 2. Materials and Methods

### 2.1. Test Location, Patient Characteristics and Data Collection Process

Surveys have been conducted among women with breast cancer to assess their quality of life, taking into account the commencement of treatment. The patients taking part in the study were women treated in Fr. B. Markiewicz Podkarpackie Oncology Centre in Brzozów, Poland, who were diagnosed with malignant breast cancer. A total of 324 women took part in the study. The following inclusion criteria were used: a histopathologically confirmed diagnosis of breast cancer, over 18 years of age, chemotherapeutic cancer treatment, and informed consent to participate in the study. The exclusion criteria were: the lack of informed patient consent to participate in the study and the occurrence of a cancer other than breast cancer within the last five years. Prior to the study, all patients were informed of its purpose, assured anonymity and voluntary participation in the study. The research was approved by the director of Fr. B. Markiewicz Podkarpackie Oncology Centre, in Brzozów, and a positive opinion was received from the Bioethics Committee (No. 47/16). The research was carried out in accordance with the principles of the Helsinki Declaration.

### 2.2. Research Tools and Measurement Techniques

The study was conducted using a diagnostic survey and the research technique used was interview. Sociodemographic data were obtained by completing a proprietary questionnaire survey. Clinical information was collected based on an analysis of medical records. The study used the prepared proprietary questionnaire and the standardized and validated European Organization for Research and Treatment of Cancer Quality of Life Questionnaire (EORTC) QLQ-C30 (quality of live questionnaire) and the module QLQ-BR23 (breast cancer) [14]. The EORTC Group has approved the use of the QLQ-C30 and QLQ-BR23 questionnaires. The QLQ-C30 questionnaire was created to assess QOL and health in various areas of patient functioning. The tool consists of 30 questions, and the answers to most of them are set on a four-point scale, i.e., not at all (1), slightly (2), significantly (3), very (4), which assess the severity of the analysed parameters. Only in the case of two questions, patients rated their health and overall QOL on a scale of 1–7 (1—very bad/bad, 7—excellent/excellent). The QLQ-C30 questionnaire is used to determine the general state of health and quality of life, as well as, in five functional scales (physical, emotional, cognitive, social, social roles), three symptomatic scales (fatigue, nausea/vomiting, pains) and six single points (questions) determining the severity of symptoms (shortness of breath, insomnia, lack of appetite, constipation, diarrhoea, and financial problems).

The EORTC QLQ-BR23 questionnaire is a complementary module to QLQ-C30 and is dedicated to patients with breast cancer. The survey consists of 23 questions, to which one answer is given on a four-point scale, i.e., not at all (1), slightly (2), significantly (3), very (4). In this case, the quality of life is assessed using functional scales (body image, sexual functioning, sexual satisfaction, future perspective) and symptom scales (altered nipples from systematic treatment, shoulder and breast ailments, hair loss).

The research results were analysed in accordance with the guidelines of the EORTC organisation [15]. The raw coefficient was calculated and then a linear transformation was performed to obtain the value of the coefficient (score), whose value for both scales and individual symptoms could be in the range from 0 to 100. A higher coefficient value for functional scales indicates a better level of functioning, and in the case of scarring scales and individual symptoms, a higher value indicates the severity (a higher level of patient distress).

In Poland, the accuracy and reliability of the QLQ-C30 questionnaire and its version BR 23 were assessed; the assessment confirmed the validity of their use in assessing the quality of life of patients with breast cancer [16]. This was one of the essential criteria when selecting a research tool to assess the quality of life.

The first surveys were conducted among women during their initial visit at a doctor after the diagnosis of symptoms while carrying out self-examination of the breast (diagnosis stage), in whom the presence of malignant breast cancer was confirmed. The women were divided into groups according to the following dates of the first visit at the doctor: ≤1 week, >1 week to 4 weeks and >4 weeks. Then, the quality of life of women was assessed at the beginning of treatment. The patients were divided into 3 groups depending on the time that passed from the diagnosis to treatment. In this way, a group of women was selected who waited for treatment ≤2 weeks; then a group of patients whose waiting time ranged from >2 weeks to 2 months, and a group of patients waiting for treatment for more than 2 months.

### 2.3. Statistical Analysis

Basic calculations of descriptive statistics were made, i.e., arithmetic mean (M), median (Me) and standard deviation (SD). The compliance of the quantitative variable distribution with the normal distribution was tested using the Shapiro–Wilk test. Due to the fact that the assumptions regarding the use of parametric methods were not met to verify statistical hypotheses, nonparametric methods were used. The statistical analysis used the Mann–Whitney U test, the Kruskal–Wallis test (together with Dunn’s post-hoc test) and the Spearman rank correlation coefficient. The results were considered statistically significant when the calculated test probability p met the inequality *p* < 0.05. Statistical analysis of test results was performed using the Statistica 10.0 software (StatSoft Inc. 2011, Crocow, Poland).

## 3. Results

### 3.1. Patient Characteristics in Terms of Sociodemographic and Medical Features

A total of 324 women treated for breast cancer were included in the study. The mean age of the patients was 52.4 years (SD = 13.7). The largest group comprised of respondents aged 41–60 (46.3%), while the share of women under 40 years of age was 24.3%, and above 60 years of age was 29.4%. The majority of patients (54.3%) lived in cities (urban populations). The study group was dominated by women living in a relationship (66.1%) and with secondary (35.2%) and higher education (33.3%). Nearly 40% of the respondents assessed their financial situation as very good, while 30.6% made the assessment as good and 29.6% as bad. Up to 41.3% of the examined respondents had their first medical consultation after finding symptoms of the disease within one week, 30.5% of the respondents had a medical consultation between after one to four weeks, and 28.1% after over four weeks. The time from confirmed diagnosis to the beginning of treatment was: for 45.1% of the respondents, up to two weeks; for 46.0% of the respondents, over two weeks up to two months; and 8.9% of respondents, waited for treatment for over two months. All patients received chemotherapy. Detailed information on sociodemographic and clinical features is presented in Table 1.

### 3.2. Overall Assessment of the Quality of Life

Based on the evaluation of individual functional scales QLQ-C30, it was found that the examined women had a reduced overall quality of life and health (M = 53.88). The following scales were rated respectively, physical (M = 74.86), social roles (M = 73.87), cognitive (M = 70.32) and social (M = 69.86). The most commonly reported symptoms were insomnia (M = 39.40), fatigue (M = 37.52), lack of appetite (M = 36.01), and nausea and vomiting (M = 33.54). The occurrence of the disease also caused financial issues in the group of women surveyed (M = 36.32). Respondents reported symptoms, such as shortness of breath, constipation and diarrhoea as of marginal importance, with the respective scale values: M = 18.11; M = 14.61; M = 10.70. The results of the quality of life assessment based on the QLQ-C30 scale are presented in Table 2.

The quality of life assessment based on the QLQ-BR23 scale is presented in Table 3. The examined women rated their body image the highest (M = 61.57), while the lowest rated parameter was sexual functioning (M = 17.49). Despite this low rating for sexual functioning, sexual satisfaction was rated at M = 46.41. Worry about the prospect of the future was rated at (M = 30.97). The respondents had the least discomfort from the breast (M = 24.25) and shoulder (M = 27.23) ailments. They also rarely pointed to side effects of the treatment (M = 31.42), while the most common symptoms were associated with stress due to hair loss (M = 68.32).

### 3.3. Spearman Rank Correlation Coefficients between QLQ-C30 Scale Values and QLQ-BR23

Positive statistically significant correlations were found between QLQ-C30 functional scales, i.e., health and quality of life, physical functioning, social roles, emotional, cognitive and social functioning, as well as body image and future perspectives (QLQ-BR23). In addition, the scale value of quality of life and health positively correlated with the scale of sexual satisfaction (QLQ-BR23). Functional scales QLQ-C30 negatively correlated with the following QLQ-BR23 scales: side effects of systematic treatment and breast and shoulder ailments. In addition, a negative correlation was found between hair loss and health status and quality of life, as well as emotional, cognitive and social functioning (Table 4).

### 3.4. Quality of Life and the Time of the First Doctor’s Visit after Discovering Disease Symptoms

A very important factor affecting the quality of life of women diagnosed with breast cancer was the elapsed time duration to visit a doctor after discovering initial disease symptoms. After collecting the necessary initial patient information, three groups of respondents were distinguished: the first, were women who after discovering symptoms of breast cancer went to consult with a doctor within one week; the second, were women who visited a doctor between after one to four weeks; and the third, were women who visited a doctor after four weeks.

The state of health and quality of life were higher in women who contacted a doctor the earliest (consultation within one week; M = 57.58), compared to women who delayed this decision (over four weeks; M = 47.8). The obtained differences were confirmed by statistical confirmation (*p* = 0.002). A similar relationship was found when assessing women’s quality of life in terms of physical, emotional and cognitive functioning. In the group of respondents who visited a doctor within one week of noticing disturbing symptoms, the mean values of these scales were significantly higher compared to the group of women who delayed their visit (over four weeks). The average values of these scales were, respectively: physical functioning—M = 78.36 vs. M = 70.99 (*p* = 0.005), emotional functioning—M = 63.43 vs. M = 52.23 (*p* = 0.004), and cognitive functioning—M = 75.37 vs. M = 62.45 (*p* = 0.000). Furthermore, in the case of performing social roles and social functioning, differences between the examined groups of women were found, but they were not confirmed statistically.

In regards to the intensity of symptoms occurring as a result of the disease, it should be emphasized that fatigue, nausea/vomiting, pain and shortness of breath were more severe in women who reported to a doctor after noticing initial disease symptoms after four weeks. On the other hand, these symptoms were less frequently reported by women who had a medical consultation within one week of noticing initial disease symptoms. The demonstrated differences between these two groups have received statistical confirmation. It was found that insomnia issues in the group of women who had a medical consultation within one week of noticing initial disease symptoms (M = 32.34) were statistically significantly smaller compared to the respondents who had a medical consultation between after one to four weeks (M = 43.10) and respondents who had a medical consultation after four weeks (M = 45.79) (*p* = 0.000). A statistically significant difference was not confirmed between the second group (medical consultation between after one to four weeks) and the third group (medical consultation after four weeks).

The time of the first visit to a doctor after observing initial disease symptoms was a factor differentiating the examined women in their lack of appetite and their financial issues. The severity of these symptoms was more intense in respondents who had a medical consultation after four weeks of noticing initial disease symptoms. Lack of appetite and financial issues were statistically significantly more intense in this group (respectively: M = 51.28; M = 46.16), compared to the group of women who had a medical consultation between after one to four weeks (respectively: M = 33.67; M = 34.34) and women who had a medical consultation within one week (respectively: M = 27.36; M = 31.09). There was no statistically significant difference regarding these symptoms between the first and second group of subjects. Detailed results of these assessments are presented in Table 5.

The values of the functional and symptomatic scales of the QLQ-BR23 questionnaire in relation to the time of a medical consultation after the initial disease symptoms had been identified are presented in Table 6. The time from the initial disease symptoms to the first medical consultation did not statistically affect the following assessments: body image, sexual functioning, sexual satisfaction, future perspectives, shoulder discomfort and hair loss. However, this variable differentiated women in assessing the side effects of systematic treatment, whose lower intensity was found in patients who had a medical consultation within one week of noticing initial disease symptoms (M = 28.5), compared to women who had a medical consultation after one to four weeks (M = 36.94). The difference showed statistical confirmation (*p* = 0.007). A similar relationship was observed in the case of ailments from the operated breast; however, due to weakly marked differences in the procedure of multiple comparisons, no statistically significant differences were found between the groups.

### 3.5. Quality of Life and Time from Confirmed Diagnosis to Treatment

The time from confirmation of the disease to the beginning of treatment affected, in many respects, the quality of life of the examined women. Among the respondents, three subgroups were distinguished, in which treatment was initiated at a different time after diagnosis, i.e., in the first group, treatment was applied within two weeks; in the second group, between two weeks and two months; and in the third group, after two months. The state of health and quality of life was rated the highest by women who initiated treatment within two weeks of confirming the diagnosis (M = 56.79), while the lowest was rated by respondents who waited longer than two months to begin treatment (M = 43.68). The shorter the time from diagnosis to treatment, the higher the quality of life was determined. The difference showed statistical confirmation (*p* = 0.019). A similar relationship was found for physical functioning. Women entering treatment within two weeks after diagnosis had higher quality of life in terms of physical function (M = 77.63), compared to patients who initiated treatment between after two weeks and two months (M = 74.09, respectively); M = 64.83; *p* = 0.023). Statistically significant differences were also shown in the area of performing social roles. Patients who waited more than two months to begin treatment after confirming diagnosis (M = 58.05) were less involved in performing social roles, compared to those who initiated treatment between after two weeks and two months (M = 72.04) and within two weeks (M = 78.88) (*p* = 0.000).

The delay in initiating treatment also resulted in a lower quality of life in terms of emotional (*p* = 0.002) and cognitive (*p* = 0.031) functioning. Statistically significant differences were confirmed between patients who initiated treatment two months after diagnosis and patients who received treatment between after two weeks and two months and those within 14 days. There were no statistically significant differences between the surveyed women in terms of social functioning. The conducted tests demonstrated the intensification of symptoms, particularly in patients who began treatment after two months from confirming diagnosis. These patients exhibited a deterioration in the quality of life due to fatigue, pain, shortness of breath, insomnia and lack of appetite. Detailed data in this regard are presented in Table 7.

Rapid implementation of treatment after diagnosis of the disease also affected the quality of life assessment expressed in the categories of QLQ-BR23 (Table 8). Body image was rated lower by women who initiated treatment after two months of confirming diagnosis (M = 54.02), compared to women who received treatment within two weeks (M = 67.72) and between after two weeks to two months (M = 61.91) from initial symptoms of the disease. However, these differences did not receive statistical confirmation. Women who began treatment after two months were more worried about their future prospects and more often noticed side effects of systematic treatment, including shoulder and breast ailments. However, only the differences between the examined women in terms of shoulder ailments were confirmed by statistically significant confirmation (*p* = 0.002). In the case of sexual functioning, no differences between the analysed groups were confirmed, while statistically significant differences in the assessment of sexual satisfaction were found. A higher value of this assessment was found in women who initiated treatment after two months (M = 77.77), compared to patients who were treated within two weeks of being diagnosed (M = 43.48) (*p* = 0.032).

## 4. Discussion

Quality of life is a subjective concept and assessed from the patient’s perspective. Current treatment of women with breast cancer should focus on effective treatments, while ensuring a high quality of life. It is particularly important to recognize the factors determining the quality of life of women with breast cancer, which may be used to specify certain actions to be taken to provide them with an adequate comfort of life.

It should be emphasized that the conducted studies dealing with important issues regarding the quality of life of women with breast cancer, in terms of time from diagnosis to treatment, have not only been the subject of research in Poland, but also abroad. Although, there are quite numerous publications on other determinants influencing women’s quality of life with breast cancer.

According to the presented research, patients diagnosed with breast cancer assessed health and quality of life at 53.88 points (SD = 19.72). The surveyed women rated their quality of life the highest in terms of physical functioning, social roles, cognitive and social functioning; however, emotional functioning was rated the lowest. The value of this scale was lower compared to the reference values—M = 61.8, given by EORTC QLQ-C30 [17]. It should be emphasized that women treated with chemotherapy were included in the presented research. In the studies of Chen et al., it was shown that the overall assessment of the quality of life and health of patients receiving chemotherapy was lower compared to those treated using other methods (51.8 vs. 55.6, respectively) [18]. Our results demonstrate that the quality of life of women was lower compared to studies conducted by Szutowicz-Wydra et al. [19], in which the quality of life of women undergoing surgery (conservative surgery, mastectomy with simultaneous breast reconstruction) was 65.0 after conservative surgery and 69.0 for women after performed mastectomy with breast reconstruction.

Our research found a reduced value on the scale for emotional functioning. The corresponding literature states that depressive states and disorders of emotional functioning are associated with, among others, young age, lack of support from relatives and a reduced economic status. Emotions of emotional functioning, expressed through sadness, apathy, depressed mood or lack of hope to overcome the disease, reduce the motivation to begin or continue long-term and comprehensive oncological treatment. Ignoring the emotional state of a patient may result in ineffective treatment being implemented, which may further result in the deterioration of the patient’s health [20,21]. The incidence of depressive states in women with breast cancer varies in various studies and populations from 1% to 50%, and it is most severe within the first six to twelve months after diagnosis [22,23].

Our study concluded, in the case of functional scales specified in the supplementary module QLQ-BR23, women assessed their body image the highest. A change in the body image occurs, among others, due to hair loss, weight change or by the surgical intervention itself, especially after a complete mastectomy. Psychological support plays a very important role during the oncological treatment process, which should be given to women at many critical moments during their treatment. The essence of empathy and a partnership approach of the medical team towards ill women should be emphasized. In a study conducted by Waldmann et al. on a group of 2366 women in Germany, who were diagnosed with breast cancer, the assessment of their own body image was the highest among the functional scales of QLQ-BR23, and also higher compared to the results obtained in this study [24]. A higher assessment of the body image made by women in the studies of Waldmann et al. could be a result of, among others, more effective psychological care and better communication between patients and medical staff. Furthermore, in the study of Trudel et al., the need for good and effective communication between cancer patients and medical staff was emphasized. These studies showed that better interpersonal communication resulted in a lower intensity of anxiety and depression in the group of patients who received oncological treatment [25].

It should be emphasized that the studies carried out in the Brzozów medical centre showed that despite the average assessment of their body image, the patients rated their sexual functioning at a very low level (17.49). The issue with the low quality of life assessment in regard to sexual functioning is often underestimated when treating women with breast cancer. Our results indicated that the assessment of sexual satisfaction among sexually active women, who took part in our study, was at a reduced level (46.41). Numerous scientific studies conducted to analyse the sexual functioning of women treated for breast cancer have shown that, in the vast majority of women, cancer and the treatment implemented affected their sexual activity and satisfaction [26,27]. Sio et al. found that the sexual functioning and satisfaction of women diagnosed with breast cancer was dependent on age. Patients over 65 years old rated their sexual functioning the lowest [28]. Our results were based on an average patient age of 52.4, which could have had an impact on the results of the sexual functioning scale.

It is considerably disturbing that the surveyed women rated their future prospects low. The value of this scale, estimated at 30.97 points, indicates that women are very anxious about their future health and proper functioning in various areas of life, as well as a possible recurrence of the disease. The low value result of this scale may be an indication that the surveyed women have problems with adapting to the new situation. The obtained results on this scale are consistent with the studies of other authors [29].

A significant conclusion of this study demonstrated that an important factor affecting the quality of life of women with breast cancer was the time of the first medical visit to a doctor after noticing initial symptoms of the disease and the time from confirming the diagnosis to initiating treatment. General health and quality of life were rated significantly higher by women who had a medical consultation within one week of noticing their first symptoms, compared to those who had a medical consultation after more than four weeks. Such differences were also found in the case of functional scales, i.e., physical, emotional and cognitive functioning. In addition, women who waited longer to see their medical practitioner experienced more severe symptoms associated with cancer, i.e., fatigue, nausea and vomiting, pain, shortness of breath, insomnia, lack of appetite and financial issues. Furthermore, the side effects of treatment were more onerous for patients who had a medical consultation only four weeks after the first symptoms of the disease.

Waiting for diagnosis and subsequent treatment increases anxiety. In the research of Villar et al., it was shown that in women who completed breast cancer treatment, their quality of life changed positively and their state of anxiety, along with its symptoms, was reduced. Therefore, it can be concluded that anxiety caused by waiting for diagnosis and treatment may reduce the quality of life of women with breast cancer [30]. In the studies of other authors, in which the quality of life of patients before and after their cancer treatment was analysed, it was determined that emotional functioning and future perspectives were rated higher after completing treatment [26,31].

Waiting for treatment had a negative impact on the quality of life of the respondents. Patients who received oncological treatment two months after diagnosis, gave the lowest assessment of their health and quality of life, including physical functioning, social roles, emotional and cognitive functioning. On the other hand, the symptoms associated with cancer (fatigue, pain, shortness of breath, insomnia, lack of appetite and shoulder discomfort on the side of the operated breast) were more burdensome for women who waited longer for treatment.

Studies conducted by Drageset et al. showed that the time that elapsed between a confirmed diagnosis and the performed breast surgery affected women’s emotional responses. Most of the examined women, while waiting for surgery, felt extreme anxiety and helplessness. They described this waiting period as long and difficult to bear. The patients expected a quickly performed surgery, which for them symbolised the beginning of a new life. Establishing the date of surgery has been shown to help reduced anxiety in women [32].

It can be assumed that initiating treatment quickly helps to not only reduce the initial shock from the diagnosis of breast cancer, but most of all, increases hope for recovery and motivates patients to fight the disease.

There are no other reports in the literature regarding the relationship between the quality of life of women with breast cancer and the time of the first visit to a doctor, as well as the waiting time for the implementation of oncological treatment in women with breast cancer. Short waiting times for doctor consultations and quickly initiated treatment contributed to a higher assessment of the quality of life expressed in the form of functional and symptomatic scales. These very important observations indicate that a medical consultation without delay and implemented treatment not only improves the prognosis in the medical context, but also significantly improves the quality of life of patients diagnosed with breast cancer. The numerous statistically significant differences found indicate the need for further, long-term research in this field, including patients from other oncological centres.

The obtained test results are particularly important due to the occurrence of delay in the diagnosis and treatment of breast cancer, which is a significant problem not only in Poland, but also around world. In the study of Jassem et al. [33], which was performed in 12 countries and covered 6588 women, it was shown that the time of delay varied from one country to another. Most delays were found among Turkish and Russian women, and then among Polish women. The average delay time due to the patient’s disregard was 4.7 weeks and the average total delay time was 14.4 weeks. Delay in cancer diagnosis due to the patient’s disregard, among others, was mainly due to: fear of diagnosis and a disregard of symptoms [34,35]. In studies conducted by Cipora et al. [36] among women with breast cancer in Poland, it was shown that the most important reasons for a delayed medical consultation were: fear of cancer diagnosis (31.2%), disregarding symptoms (19.5%), long waiting time for medical consultation (13.9%), fear of necessary radical treatment (8.7%), lack of time (6.9%), fear of losing employment (3.9%), use of unconventional methods (2.6%) and poor financial situation (2.2%).

Studies conducted by Visser et al. [37] assessed the quality of life of recently diagnosed patients with lung, pancreatic, esophagus and cervical cancer who were awaiting surgery. It was shown that recently diagnosed cancer patients waiting for surgery suffered from mental disorders and their quality of life was reduced. Comparably, our study results determined, patients who waited the longest time for treatment (over two months) had a diminished quality of life compared to those who received treatment within two weeks.

In summary, the waiting period for treatment should be as short as possible, which determines the medical prognosis but is also important due to the higher quality of life assessment. During the waiting time for treatment, a significant role should be carried out mainly by doctors, nurses, psychologists and all practitioners who provide emotional support, lifestyle advice and provide simple treatment for symptoms, such as fatigue, a depressed mood and insomnia.

## 5. Conclusions

1. The overall assessment of the state of health and quality of life of women with breast cancer decreased. Women had a reduced level of QOL in their emotional and sexual spheres of life and also rated their future prospects negatively. These factors should be taken into account when planning psychotherapy and the doctor–patient relationship.
2. The quality of life of the examined women depended on the time of initiated treatment. Patients had a better quality of life who had a medical consultation the earliest after noticing initial symptoms of the disease and those whose waiting time for treatment was shorter.
3. The time interval between the individual stages of diagnosis and initiated treatment in breast cancer patients should be as short as possible, preferably in a systematic way. Medical practitioners should strive to diagnose and treat all such patients in breast oncology units.
4. There is a need for further research and discussion on the relationship between the quality of life of patients and the occurrence of diagnostic and therapeutic delays related to breast cancer, as the conclusions implemented in practice may improve the quality of life of ill women.

## Figures and Tables

**Table 1:** Patient characteristics in terms of sociodemographic and medical (clinical) features.

Feature	N	%
**Age (average 52,4, SD = 13,7)** **Min-Max 26-75 y.o.a**		
<40	79	24.3
41–60	150	46.3
>60	95	29.4
**Dwelling place**		
Rural	148	45.7
Urban	176	54.3
**Marital status**		
Single	110	33.9
In relationship	214	66.1
**Education**		
Vocational	102	31.5
Secondary	114	35.2
Higher	108	33.3
**Financial situation**		
Very good	129	39.8
Good	99	30.6
Bad	96	29.6
**Menopausal status**		
Pre-menopausal	154	47.6
Post- menopausal	170	52.4
**Type of treatment**		
Chemotherapy	324	100
**The date of the first visit to the doctor after finding out the symptoms of the disease**		
≤1 week	134	41.3
>1 week up to 4 weeks	99	30.5
>4 weeks	91	28.2
**Time from confirmation of the diagnosis to taking up treatment**		
≤2 weeks	146	45.1
>2 weeks up to 2 months	149	46.0
>2 months	29	8.9

y.o.a – years of age.

**Table 2:** Assessment of women’s quality of life—categories related to QLQ-C30.

QLQ-C30	M	Me	SD
**Health status and quality of life**	53.88	50.00	19.72
Functional scales *			
Physical functioning	74.86	80.00	18.07
Performing social roles	73.87	66.67	22.89
Emotional functioning	59.77	66.67	24.99
Cognitive functioning	70.32	66.67	25.52
Social functioning	69.86	66.67	28.68
The scale of the symptoms **			
Fatigue	37.52	33.33	20.07
Nausea/vomiting	33.54	16.67	37.26
Pain	24.28	16.67	20.66
Shortness of breath	18.11	0.00	24.35
Insomnia	39.40	33.33	27.77
Lack of appetite	36.01	33.33	33.17
Constipation	14.61	0.00	23.00
Diarrhoea	10.70	0.00	18.42
Financial problems	36.32	33.33	32.47

M—arithmetic mean, Me—median, SD—standard deviation; * a higher value means a better level of functioning and quality of life (min. 0, max. 100). ** a higher value means a greater severity of symptoms (min. 0, max. 100).

**Table 3:** Assessment of women’s quality of life—categories related to QLQ-BR23.

QLQ-BR23	M	Me	SD
Functional Scales *			
An image of your own body	61.57	66.67	32.95
Sexual function	17.49	0.00	23.56
Sexual satisfaction	46.41	33.33	28.59
The prospect of the future	30.97	33.33	33.86
The scale of the symptoms **			
Side effects of systemic treatment	31.42	28.57	21.25
Ailments related to the arm	27.23	22.22	19.18
Ailments related to the breast	24.25	25.00	18.89
Hair loss	68.32	66.67	35.78

M—arithmetic mean, Me—median, SD—standard deviation; * a higher value means a better level of functioning and quality of life (min. 0, max. 100). ** a higher value means a greater severity of symptoms (min. 0, max. 100).

**Table 4:** Spearman rank correlation coefficients between the QLQ-C30 and QLQ-BR23 scale values *.

	An Image of Your Own Body	Sexual Function	Sexual Satisfaction	The Prospect of the Future	Side Effects of Systemic Treatment	Ailments Related to the Arm	Ailments Related to the Breast	Hair Loss
**Health status and quality of life**	0.289 *	0.085	0.276 *	0.212 *	-0.374 *	-0.298 *	-0.311 *	-0.263 *
**Physical functioning**	0.284 *	0.064	0.106	0.186 *	−0.351 *	−0.310 *	−0.227 *	−0.032
**Performing social roles**	0.253 *	−0.003	−0.082	0.118 *	−0.278 *	−0.348 *	−0.272 *	−0.112
**Emotional functioning**	0.336 *	0.071	0.139	0.286 *	−0.510 *	−0.243 *	−0.359 *	−0.276 *
**Cognitive functioning**	0.317 *	0.005	0.175	0.234 *	−0.440 *	−0.239 *	−0.337 *	−0.241 *
**Social functioning**	0.380 *	−0.076	0.048	0.198 *	−0.320 *	−0.198 *	−0.253 *	−0.181 *
**Fatigue**	−0.366 *	−0.073	−0.033	−0.336 *	0.508 *	0.343 *	0.318 *	0.163 *
**Nausea/vomiting**	−0.241 *	0.009	−0.218 *	−0.145 *	0.617 *	0.285 *	0.399 *	0.256 *
**Pain**	−0.289 *	−0.027	−0.096	−0.181 *	0.430 *	0.458 *	0.356 *	0.097
**Shortness of breath**	−0.223 *	−0.017	−0.017	0.027	0.278 *	0.306 *	0.308 *	−0.024
**Insomnia**	−0.307 *	−0.118*	−0.105	−0.275 *	0.464 *	0.241 *	0.282 *	0.123
**Lack of appetite**	−0.241 *	−0.037	−0.107	−0.205 *	0.549 *	0.362 *	0.419 *	0.121
**Constipation**	−0.149 *	−0.075	−0.082	−0.106	0.237 *	0.123 *	0.113 *	0.040
**Diarrhoea**	−0.236 *	0.132 *	0.009	−0.046	0.288 *	0.123 *	0.126 *	0.149 *
**Financial problems**	−0.235 *	−0.064	−0.009	−0.184 *	0.400 *	0.168 *	0.251 *	0.143

* statistically significant relationships.

**Table 5:** Assessment of women’s quality of life—categories related to QLQ-C30 and the time of the first doctor’s visit after discovering disease symptoms.

QLQ-C30	*p*	The Time of the First Doctor’s Visit after Discovering Disease Symptoms								
		within 1 Week *n* = 134			above 1 Week up 4 Weeks *n* = 99			above 4 Weeks *n* = 91		
		M	Me	SD	M	Me	SD	M	Me	SD
**Health status and quality of life**	0.002	57.58 ^a^	58.33	20.19	54.21	50.00	19.02	47.80 ^b^	50.00	18.42
**Functional Scales ^1^**										
Physical functioning	0.005	78.36 ^a^	80.00	16.68	73.67	80.00	18.10	70.99 ^b^	73.33	19.20
Performing social roles	0.019 *	77.24	83.33	23.62	71.55	66.67	21.20	71.43	66.67	23.21
Emotional functioning	0.004	63.43 ^a^	66.67	24.05	61.70	66.67	24.42	52.23 ^b^	66.67	25.61
Cognitive functioning	0.000	75.37 ^a^	83.33	24.67	70.70	66.67	23.94	62.45 ^b^	66.67	26.71
Social functioning	0.484	72.89	66.67	25.25	68.69	66.67	30.70	66.67	66.67	30.93
**The scale of the symptoms ^2^**										
Fatigue	0.008	34.41 ^a^	33.33	20.66	37.49	33.33	20.55	42.12 ^b^	33.33	17.88
Nausea/vomiting	0.000	23.38 ^a^	0.00	31.00	34.17	16.67	37.37	47.80 ^b^	33.33	40.99
Pain	0.002	20.02 ^a^	16.67	19.58	25.59	16.67	20.94	29.12 ^b^	33.33	20.87
Shortness of breath	0.0005	12.44 ^a^	0.00	20.71	20.54	0.00	26.81	23.90 ^b^	33.33	24.99
Insomnia	0.0001	32.34 ^a^	33.33	29.17	43.10 ^b,c^	33.33	27.04	45.79 ^c^	33.33	24.16
Lack of appetite	0.0000	27.36 ^a,b^	33.33	30.27	33.67 ^b^	33.33	31.76	51.28 ^c^	33.33	33.81
Constipation	0.509	13.93	0.00	23.23	14.48	0.00	24.36	15.75	0.00	21.28
Diarrhoea	0.185	8.96	0.00	18.36	11.11	0.00	16.50	12.82	0.00	20.35
Financial problems	0.003	31.09 ^a,b^	33.33	28.96	34.34 ^b^	33.33	33.82	46.16 ^c^	33.33	33.92

M—arithmetic mean, Me—median, SD—standard deviation * no statistically significant differences were found in the post hock analysis. ^1^ higher value means a better level of functioning and quality of life (min. 0, max. 100). ^2^ higher value means greater severity of symptoms (min. 0, max. 100). ^a, b, c^—mean values in different letters differ statistically significantly at *p* < 0.05.

**Table 6:** Assessment of women’s quality of life—categories related to QLQ-BR23 and the time of the first doctor’s visit after discovering disease symptoms.

QLQ-BR23	*p*	The Time of the First Doctor’s Visit after Discovering Disease Symptoms								
		within 1 Week *n* = 134			above 1 Week up to 4 Weeks *n* = 99			above 4 Weeks *n* = 91		
		M	Me	SD	M	Me	SD	M	Me	SD
**Functional Scales ^1^**										
An image of your own body	0.369	61.94	66.66	29.55	59.00	66.67	33.60	63.83	75.00	36.92
Sexual function	0.407	19.03	0.00	26.19	18.18	16.67	21.57	14.47	0.00	21.40
Sexual satisfaction	0.304	50.00	50.00	31.23	40.40	33.33	21.66	47.83	33.33	31.50
The prospect of the future	0.183	33.83	33.33	33.70	31.31	33.33	33.61	26.73	0.00	34.25
**The scale of the symptoms ^2^**										
Side effects of systemic treatment	0.007	28.50 ^a^	23.81	20.48	30.30	28.57	21.40	36.94 ^b^	33.33	21.94
Ailments related to the arm	0.552	25.54	22.22	20.53	27.72	22.22	21.14	29.18	33.33	14.21
Ailments related to the breast	0.048 *	23.07	16.67	20.05	23.06	16.67	18.93	27.29	25.00	16.85
Hair loss	0.126	69.70	83.33	34.50	74.69	100	33.61	61.29	66.67	37.79

M—arithmetic mean, Me—median, SD—standard deviation; * no statistically significant differences were found in the post hock analysis. ^1^ higher value means a better level of functioning and quality of life (min. 0, max. 100). ^2^ higher value means greater severity of symptoms (min. 0, max. 100). ^a, b^ mean values in different letters differ statistically significantly at *p* < 0.05.

**Table 7:** Assessment of women’s quality of life—categories related to QLQ-C30 and the time from diagnosis confirmation to treatment.

QLQ-C30	*p*	Time from Confirmation of the Diagnosis to Treatment								
		up to 2 Weeks *n* = 146			above 2 Weeks up to 2 Months *n* = 149			above 2 Months *n* = 29		
		M	Me	SD	M	Me	SD	M	Me	SD
**Health status and quality of life**	0.019	56.79 ^a^	58.33	18.90	53.02	50.00	19.09	43.68 ^b^	50.00	24.36
**Functional Scales ^1^**										
Physical functioning	0.023	77.63 ^a^	80.00	16.23	74.09 ^b.c^	73.33	16.47	64.83 ^c^	73.33	28.56
Performing social roles	0.000	78.88 ^a^	83.33	21.73	72.04 ^b.c^	66.67	20.80	58.05 ^c^	66.67	30.08
Emotional functioning	0.002	60.67 ^a.b^	66.67	25.00	62.02 ^b^	66.67	23.85	43.68 ^c^	50.00	25.65
Cognitive functioning	0.031	71.35 ^a.b^	66.67	25.58	71.92 ^b^	83.33	23.74	56.9 ^c^	66.67	30.71
Social functioning	0.619	71.00	66.67	25.97	70.69	66.67	28.58	59.77	66.67	39.47
**The scale of the symptoms ^2^**										
Fatigue	0.006	35.46 ^a.b^	33.33	18.83	36.76 ^b^	33.33	19.07	51.72 ^c^	44.44	25.59
Nausea/vomiting	0.052	30.82	16.67	36.90	32.30	16.67	36.78	50.00	50.00	40.09
Pain	0.000	20.21 ^a.b^	16.67	19.30	24.72 ^b^	16.67	18.44	42.53 ^c^	33.33	27.67
Shortness of breath	0.000	12.10 ^a^	0.00	20.67	20.81 ^b.c^	0.00	23.44	35.63 ^c^	33.33	34.42
Insomnia	0.001	35.53 ^a.b^	33.33	27.28	38.48 ^b^	33.33	25.91	58.62 ^c^	66.67	30.41
Lack of appetite	0.029	32.19 ^a^	33.33	32.32	36.91	33.33	32.22	50.57 ^b^	66.67	37.40
Constipation	0.181	13.70	0.00	23.05	13.20	0.00	19.30	26.44	0.00	34.94
Diarrhoea	0.963	9.82	0.00	15.25	10.74	0.00	19.09	14.94	0.00	27.58
Financial problems	0.093	32.88	33.33	29.29	36.69	33.33	32.59	51.72	33.33	42.34

M—arithmetic mean, Me—median, SD—standard deviation; ^1^ higher value means a better level of functioning and quality of life (min. 0, max. 100). ^2^ higher value means greater severity of symptoms (min. 0, max. 100). ^a, b, c^ mean values in different letters differ statistically significantly at *p* < 0.05.

**Table 8:** Assessment of women’s quality of life—categories related to QLQ-BR23 and the time from diagnosis confirmation to treatment.

QLQ-BR23	*p*	Time from Confirmation of the Diagnosis to Treatment								
		up to 2 Weeks *n* = 146			above 2 Weeks up to 2 Months *n* = 149			above 2 Months *n* = 29		
		M	Me	SD	M	Me	SD	M	Me	SD
**Functional Scales ^1^**										
An image of your own body	0.555	67.72	66.67	31.83	61.91	66.67	33.14	54.02	50.00	37.51
Sexual function	0.738	15.56	0.00	23.20	18.34	0.00	23.23	17.82	0.00	27.43
Sexual satisfaction	0.032	43.48 ^a^	33.33	27.10	45.33	33.33	28.38	77.77 ^b^	83.33	27.22
The prospect of the future	0.428	29.45	33.33	33.11	33.33	33.33	34.44	26.44	0.00	34.94
**The scale of the symptoms ^2^**										
Side effects of systemic treatment	0.225	31.60	28.57	20.59	29.75	28.57	20.56	39.08	38.09	26.52
Ailments related to the arm	0.002	24.05 ^a^	22.22	19.62	29.60 ^b^	33.33	16.97	31.03	22.22	25.09
Ailments related to the breast	0.024 *	21.92	16.67	18.28	25.11	25.00	18.76	31.61	33.33	20.94
Hair loss	0.086	72.94	100	36.19	66.24	66.67	35.02	56.14	33.33	35.23

M—arithmetic mean, Me—median, SD—standard deviation; * no statistically significant differences were found in the post hock analysis. ^1^ higher value means a better level of functioning and quality of life (min. 0, max. 100). ^2^ higher value means greater severity of symptoms (min. 0, max. 100). ^a, b^ mean values in different letters differ statistically significantly at *p* < 0.05.
